# Development of a novel peptide inhibitor of subtilisin BPN′

**DOI:** 10.1002/2211-5463.13481

**Published:** 2022-09-22

**Authors:** Kohki Ishida, Makoto Shimizu, Ayumi Wakasugi, Yuko Matsui, Akira Nakamura, Shuichi Kojima

**Affiliations:** ^1^ Department of Life Science, Graduate School of Science Gakushuin University Tokyo Japan; ^2^ Department of Chemistry, Graduate School of Science Gakushuin University Tokyo Japan; ^3^ Department of Chemistry, Faculty of Science Gakushuin University Tokyo Japan; ^4^ Department of Life Science, Faculty of Science Gakushuin University Tokyo Japan

**Keywords:** disulfide bond, inhibitory peptide, phage display, protease inhibitor, protein–peptide interactions, subtilisin BPN′

## Abstract

Proteinaceous protease inhibitors can strongly and specifically inhibit cognate proteases, but their use as pharmaceuticals is limited by their size. As such, the development of effective protease peptide inhibitors would be beneficial for biochemical studies and drug discovery. In this study, we applied a phage display system to select subtilisin BPN′‐binding peptides and evaluated their inhibitory activities against subtilisin BPN′. A 12mer peptide with an intramolecular disulfide bond inhibited subtilisin BPN′ (*K*_i_ value of 13.0 nm). Further mutational analyses of the peptide resulted in the development of a short peptide inhibitor against subtilisin BPN′ that showed high inhibitory activity and binding affinity (*K*_i_ value of 0.30 nm). This activity was found to be derived from the conformational rigidity caused by the intramolecular disulfide bond and the small residue at the P1′ site and from the interaction of the P4 and P6′ residues with subtilisin BPN′.

## Graphical abstract

We identified several potential inhibitory peptides for subtilisin BPN′, a typical serine protease, by phage display method. Of these, a 12mer peptide, named ASIP, showed inhibitory activity with an inhibition constant (*K*_i_) of 13 nm against subtilisin BPN′. Subsequent mutation analyses on this peptide led to the development of an improved inhibitory peptide with *K*_i_ of 0.3 nm.

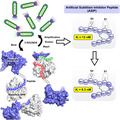

**Abbreviations**
ASIP: artificial subtilisin inhibitor peptide
CI‐2: hiproly barley chymotrypsin inhibitor 2
EGL: leech eglin‐C
MST: marinostatin
OMTKY3: turkey ovomucoid third domain
SSI: *Streptomyces* subtilisin inhibitor
TI‐II: wound‐induced tomato protease inhibitor‐II

Protein–protein interactions are involved in various cellular processes, such as signal transduction, electron transfer, protein modification, cellular metabolism, and immune system functions. Protein functions including enzyme activities can be modulated via complex formation through protein–protein interactions. Among these, the interactions between proteases and their inhibitors have been well studied for many decades. In addition, short peptides, including cyclic forms, are attractive compounds for drug discovery because they regulate disease‐related protease functions [1].

Proteases are enzymes that catalyze the hydrolysis of peptide bonds in peptides and proteins. Serine proteases are the most studied protease family, and they have the Ser‐His‐Asp catalytic triad in their active site and are widely involved in biological processes. For example, matriptase is associated with cancer cell metastasis [2]. Therefore, the development of protease inhibitors might be applicable for pharmaceutical purposes. For example, inhibitors targeting a critical cysteine protease of severe acute respiratory syndrome coronavirus 2 (SARS‐CoV‐2) have been developed to inhibit SARS‐CoV‐2 viral replication [3, 4].

Numerous inhibitors of serine proteases have been discovered in nature or developed through chemical synthesis, and they have a wide size distribution from small molecules to proteins. Peptides could stand out as inhibitors because of their variability, productivity, and efficacy. Proteinaceous protease inhibitors can enhance the interaction with proteases or alter the specificity of proteases through the introduction of amino acids into the reactive site loops [5, 6, 7]. Proteinaceous inhibitors can also strongly and specifically inhibit cognate proteases, although they are less effective than pharmaceuticals because their size limits their productivity. The synthesis of small molecules with various functional groups is generally easy; however, the interactions between these molecules and cognate proteases are less effective compared with those of proteinaceous inhibitors. Therefore, the development of peptide inhibitors with the same strong inhibitory activity as proteinaceous inhibitors represents an attractive goal not only because the synthesis is easier but also because such work could reveal the underlying inhibitory mechanisms.

Phage display is a powerful screening method to detect interactions between a target molecule (e.g., a protein) and a peptide or protein genetically inserted into a coat protein gene of a bacteriophage [8]. In combination with molecular evolutionary engineering techniques, phage display has been used to enhance the interaction and specificity of epitopes for antibodies [9] and ligands for receptors [10]. In this study, the phage display method was used to identify a novel functional peptide that inhibits the typical bacterial serine protease subtilisin BPN′. The peptide inhibitor was composed of 12 amino acid residues, which is the length of the smallest subtilisin inhibitor marinostatin [11]. Furthermore, by introducing amino acid substitutions, we created an improved subtilisin inhibitor peptide with a sub‐nanomolar inhibition constant.

## Materials and methods

### Materials

Subtilisin BPN′ and *N*‐succinyl‐Ala‐Ala‐Pro‐Phe‐*p‐*nitroanilide (*N*‐suc‐AAPF‐*p*NA) were purchased from Sigma‐Aldrich (Tokyo, Japan). The oligopeptides used in this study were synthesized by Eurofins Genomics (Tokyo, Japan). The formation of an S‐S bond in each peptide was accomplished by incubating an equimolar amount of cystine with the peptide, and the concentrations of the oxidized peptides were determined by amino acid analyses after hydrolysis of the peptide with 6 m HCl at 110 °C for 24 h *in vacuo*.

### Phage display and selection of peptides

Phage display was performed using a Ph.D.‐12 Phage Display Peptide Library Kit (New England Biolabs, Tokyo, Japan). According to the protocol, phages were co‐incubated with biotin‐labeled subtilisin BPN′, and then, the mixture was poured onto a streptavidin plate. The plate was washed to remove nonspecifically bound phages, and phages specifically bound to subtilisin BPN′ were amplified. After three rounds of panning, the phages were collected. The nucleotide sequences of the selected phages were determined by dideoxy sequencing of phage DNA.

### Measurements of inhibitory activities and determination of inhibition constants (*K*_i_) of peptides

The inhibitory activities of peptides against subtilisin BPN′ were preliminarily measured using the casein‐Folin method. First, casein was incubated with subtilisin BPN′ in the absence or presence of various molar ratios of synthetic peptides at 37 °C and pH 7.0 for 10 min. Trichloroacetic acid was added to the reaction mixture to precipitate the undigested casein, and the supernatant was mixed with sodium carbonate and Folin's phenol reagent. The absorbance at 660 nm was then measured to determine the amount of peptide obtained by digestion. Inhibitory activity was defined as the reduction in the amount of peptides derived from the enzymatic digestion of casein in the presence of a synthetic peptide when compared to the amount of peptides in the absence of synthetic peptides. To determine the *K*_i_ values, the proteolytic activities of subtilisin BPN′ in the presence of various molar ratios of synthetic peptides were measured using a synthetic substrate, *N*‐suc‐AAPF‐*p*NA, as described previously [12], and then, the *K*_i_ values were calculated by nonlinear curve fitting using the Morrison equation [13].

## Results

### Screening of subtilisin‐binding peptides

Based on the phage display experiment with three cycles of panning, eight 12‐residue sequences were identified from the 16 phages analyzed (Table 1). Among these, phages that displayed a peptide with the amino acid sequence Ser‐Asp‐Phe‐Ser‐Cys‐Leu‐Ser‐Glu‐Gly‐Cys‐Arg‐Thr (peptide I) were found seven times, implying that this peptide displayed on the coat proteins of phages shows the highest affinity to subtilisin BPN′ and might have strong inhibitory activities against subtilisin BPN′. Therefore, we designated peptide I as an artificial subtilisin inhibitor peptide (ASIP), and ASIP was used as a prototype for the subsequent mutational study.

**Table 1:** Amino acid sequences of peptides selected by phage display.

Name	Count	1	2	3	4	5	6	7	8	9	10	11	12
I	7	Ser	Asp	Phe	Ser	Cys	Leu	Ser	Glu	Gly	Cys	Arg	Thr
II	2	His	Asp	Phe	Thr	Cys	Leu	Ser	Val	Phe	Cys	Phe	Pro
III	2	Val	Pro	Trp	Trp	Lys	His	Pro	Pro	Leu	Pro	Val	Pro
IV	1	His	Val	Lys	Trp	Pro	His	Trp	Trp	Asp	Lys	Arg	Pro
V	1	Asp	Glu	Leu	Arg	Cys	Leu	Ala	Ser	Trp	Cys	Lys	Ile
VI	1	Phe	Asn	Ser	Gln	Leu	Pro	Ser	His	Arg	Leu	Ala	Arg
VII	1	Leu	Asp	Leu	Pro	Arg	Cys	Leu	Val	Pro	Ala	Leu	Cys
VIII	1	Ser	Pro	Ser	Phe	Gln	Ile	Leu	Ser	Pro	Tyr	Pro	Leu

### Characterization of ASIP

Since the sequence of ASIP was the most frequently selected in the phage display experiments, we intended to characterize its inhibitory properties with subtilisin BPN′ in solution using chemically synthesized ASIP. Two cysteine residues were identified in ASIP, which indicates that a disulfide bond might be formed in this peptide between Cys5 and Cys10. To clarify the relationship between disulfide bond formation and its activity as a subtilisin inhibitor, we measured the inhibitory activities of ASIP with or without a disulfide bond. ASIP without a disulfide bond not only lacked inhibitory activity against subtilisin BPN′ but was also specifically cleaved at the ASIP peptide bond between Leu6 and Ser7 to produce two peptides with sequences of Ser‐Asp‐Phe‐Ser‐Cys‐Leu and Ser‐Glu‐Gly‐Cys‐Arg‐Thr, indicating that Leu6 of ASIP is considered the P1 position residue of the inhibitor. By incubating reduced ASIP with equimolar cystine, the resultant ASIP quantitatively formed the intramolecular disulfide bond, which was confirmed by reverse‐phase HPLC and matrix‐assisted laser desorption‐ionization time‐of‐flight mass spectrometry (data not shown). The inhibitory properties of the oxidized form of ASIP were then investigated. Oxidized ASIP clearly exhibited inhibitory activity toward the digestion of a synthetic substrate of subtilisin in a concentration‐dependent manner, and the inhibition constant was determined to be 13.0 nm. These results suggest that the disulfide bond formed between Cys5 and Cys10 is essential for subtilisin inhibition.

### Inhibitory activity of truncated derivatives of ASIP

**Fig. 1:**
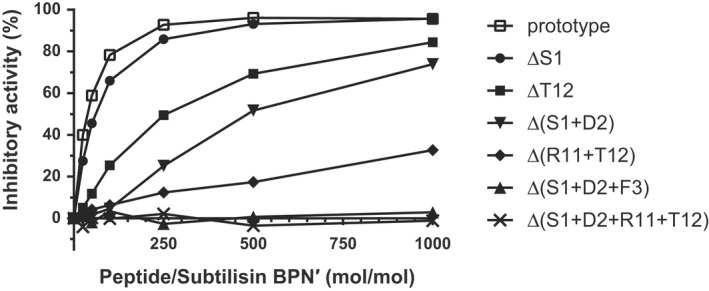
Inhibition of subtilisin BPN′ by deletion mutants of ASIP. The inhibitory activity, determined by the casein‐Folin method, was plotted against the molar ratio of the ASIP peptides to subtilisin BPN′. The solid lines with open squares, circles, filled squares, inverted triangles, rhombuses, triangles, and crosses indicate the prototype, ΔS1, ΔT12, Δ(S1 + D2), Δ(R11 + T12), Δ(S1 + D2 + F3), and Δ(S1 + D2 + R11 + T12), respectively.

To investigate the required minimum length of ASIP for strong inhibition of subtilisin BPN′, several truncated derivatives of ASIP were constructed and their inhibitory activities toward subtilisin BPN′ were preliminarily measured using casein as a substrate. However, the inhibitory activities obtained by this method were generally lower than those obtained using a synthetic substrate. ΔS1, Δ(S1 + D2), and Δ(S1 + D2 + F3) represent peptides truncated by one, two, and three residues from the N‐terminus of ASIP, respectively, and ΔT12 and Δ(R11 + T12) represent peptides truncated by one and two residues from the C‐terminus, respectively. As shown in Fig. 1, the inhibitory activity of ΔS1 against subtilisin BPN′ was slightly lower than that of ASIP, although it was in nearly the same range as that with high inhibitor/protease ratios. The inhibitory activity of Δ(S1 + D2) was approximately 23% lower than that of ASIP at an inhibitor:protease molar ratio of 1000 : 1. These results indicate that the two N‐terminal residues (Ser1 and Asp2) do not interact strongly with subtilisin BPN′. However, the C‐terminal truncations ΔT12 and Δ(R11 + T12) exhibited decreased inhibitory activities (12% and 66% lower than that of ASIP, respectively) compared to those of the N‐terminal truncations, with the decrease corresponding to the length of the truncation. The inhibitory activity of ASIP was affected by the C‐terminal truncations, rather than the N‐terminal truncations, implying that the C‐terminal region of ASIP makes a larger contribution to the binding and inhibition of subtilisin BPN′. The truncation of both ends, namely Δ(S1 + D2 + R11 + T12), resulted in a complete loss of inhibitory activity. Taken together, the amino acid region Asp2‐Thr12 is necessary for the significant inhibition of subtilisin activity. In addition, Δ(S1 + D2 + F3) almost completely lost its inhibitory activity, suggesting that the bulky residue Phe3 likely acts as the P4 residue of inhibitors that fit into the large S4 pocket of subtilisin BPN′. Although at least 11 residues are required for strong binding between a peptide and subtilisin BPN′, subsequent mutational analyses were carried out using the 12‐residue peptide to ensure that the tight binding of the peptide with subtilisin BPN′ could be maintained.

### Alanine scanning of ASIP

**Fig. 2:**
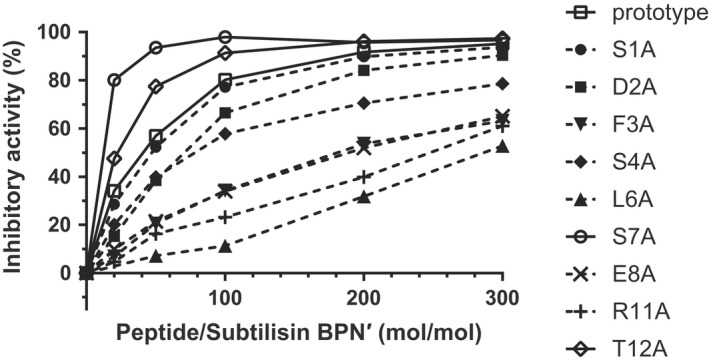
Inhibition of subtilisin BPN′ by single‐point mutants of ASIP. The inhibitory activity, determined by the casein‐Folin method, was plotted against the molar ratio of the ASIP peptides to subtilisin BPN′. The solid lines with open circles, rhombuses, and squares indicate S7A, T12A, and the prototype, respectively. The dashed lines with filled circles, squares, inverted triangles, rhombuses, triangles, crosses, and plus marks represent S1A, D2A, F3A, S4A, L6A, E8A, and R11A, respectively.

To evaluate the contribution of each amino acid of ASIP to its inhibitory activity, a single‐alanine scanning experiment for ASIP was carried out (except for Cys5, Gly9, and Cys10), and then, the inhibitory activities were measured using casein as a substrate similar to that with the truncated ASIP. As shown in Fig. 2, the inhibitory activity of Ser1Ala (S1A) was comparable to that of the prototype ASIP, which was consistent with the results for the ΔS1 derivative. Thus, the hydroxy group in the side chain of the 1st residue of ASIP is not involved in the interaction between the inhibitor and subtilisin BPN′. The inhibitory activities of D2A and R11A were also similar to those of the truncated derivatives Δ(S1 + D2) and Δ(R11 + T12), respectively, although the single amino acid substitutions had relatively small effects compared with those of the truncation. Among the mutants, the alanine substitutions at Ser7 (S7A) and Thr12 (T12A) led to increased inhibitory activities, whereas the substitutions at the other residues resulted in decreased inhibitory activity. These results suggest that the S7A or T12A mutant of ASIP is a more potent inhibitor of subtilisin BPN′ than the prototype ASIP and indicate that the peptide with the sequence selected by phage display does not always show the strongest binding ability, which might be related to the restricted number of mutants displayed on the coat protein.

F3A, L6A, E8A, and R11A showed relatively weaker inhibitory activities than the prototype ASIP. In particular, Leu6 and Phe3 were presumed to be the P1 and P4 residues, respectively, as indicated by the specific cleavage of the linear form of ASIP between Leu6 and Ser7 by subtilisin BPN′. The side chains of these residues were thought to be likely responsible for the strong binding of ASIP to subtilisin BPN′. Moreover, Glu8 and Arg11 were assumed to form salt bridges or hydrogen bonds with subtilisin BPN′ via polar side chains.

### Amino acid substitutions at Ser7 and Thr12

To examine the possibility of improving the inhibitory activity and binding ability of ASIP to subtilisin BPN′, we focused on Ser7 and Thr12, which showed increased inhibitory activity in alanine scanning experiments. These two residues were substituted with various amino acids, and their inhibitory activities were evaluated by determining the *K*_i_ values for subtilisin BPN′ using *N*‐suc‐AAPF‐*p*NA as a synthetic substrate. As shown in Table 2, the prototype ASIP exhibited a *K*_i_ value of 13.0 nm. The S7A and S7G substitutions showed stronger binding and inhibition (*K*_i_ = 2.58 and 5.14 nm, respectively), whereas the S7V, S7F, S7D, and S7K mutations significantly decreased inhibition. These results suggest that a residue with a large side chain is inappropriate at the P1′ position, even if the residue is hydrophobic or hydrophilic, which is probably because of steric hindrance in binding with subtilisin BPN′.

**Table 2:** Inhibition constants of ASIP‐derived peptides. ND, not detected.

	*K*_i_ (nm)		*K*_i_ (nm)		*K*_i_ (nm)
Prototype	12.97 ± 1.64				
S7A	2.58 ± 0.20	T12A	17.39 ± 1.42	S7A + T12A	2.72 ± 0.04
S7G	5.14 ± 0.30	T12G	10.58 ± 0.55	S7G + T12G	7.73 ± 0.34
S7V	ND	T12L	12.92 ± 1.02	S7A + T12K	0.30 ± 0.02
S7D	ND	T12D	23.13 ± 3.35		
S7K	ND	T12K	5.43 ± 0.29		
S7F	ND	T12R	4.41 ± 0.24		

For Thr12, the replacement with alanine (T12A) resulted in a slight decrease in inhibitory activity (*K*_i_ = 17.4 nm) compared with that of the prototype ASIP, although T12A showed slightly increased inhibition of subtilisin when casein was used. In addition, the *K*_i_ values of T12G and T12L were calculated as 10.6 and 12.9 nm, respectively, indicating that the substitution of Thr12 with uncharged residues did not significantly improve the inhibitory activity. By contrast, drastic changes in the inhibitory activity were obtained by substituting charged residues. T12K and T12R showed significantly higher inhibition and binding affinity, as shown by the *K*_i_ value, which presented as a more than a two‐fold reduction, whereas T12D decreased the inhibitory activity compared to that of the prototype (Thr at the 12^th^ position). These results indicate that a basic residue at the 12^th^ position of ASIP enhances the inhibitory activity against subtilisin BPN′, which might be due to an electrostatic interaction between the positively charged C‐terminal residue of the inhibitor peptide and the negatively charged surface of subtilisin BPN′.

The combination of mutations at the 7^th^ and 12^th^ positions was also examined. S7A + T12A and S7G + T12G had similar inhibitory activities to those of S7A and S7G, respectively, in which the substitution of Thr12 with Ala and Gly did not significantly affect binding. However, S7A + T12K showed the highest inhibitory activity and binding affinity, with a *K*_i_ value of 0.30 nm, which indicated that S7A + T12K inhibited subtilisin BPN′ approximately 40‐fold more than the prototype ASIP. Such a high inhibitory activity of S7A + T12K might be due to a synergistic effect of combining single substitutions because each substitution increased the inhibitory activity of the prototype ASIP, with a 5‐fold increase with the S7A substitution and a 2.4‐fold increase with the T12K substitution according to their *K*_i_ values.

## Discussion

The results showed that the linear form of the chemically synthesized peptide (ASIP) with a sequence selected by the phage display system was specifically cleaved at Leu6‐Ser7 by subtilisin BPN′ whereas the oxidized form of the peptide with an S‐S bond between Cys5 and Cys10 inhibited subtilisin BPN′. These findings indicate that the P1 residue of ASIP is Leu6, which is consistent with the substrate specificity of subtilisin BPN′, and suggest that the rigid conformation around the P1 residue caused by the S‐S bond is required for the inhibitory activity of ASIP, which is consistent with many other proteinaceous inhibitors. The binding ability of the prototype ASIP was subsequently increased 40‐fold based on the Ser7 → Ala and Thr12 → Lys mutations. Then, we compared the amino acid sequence of the resulting strong inhibitor with that of other subtilisin inhibitors for which the three‐dimensional structures are known (e.g., turkey ovomucoid third domain (OMTKY3) [14], hiproly barley chymotrypsin inhibitor 2 (CI‐2) [15], wound‐induced tomato protease inhibitor‐II (TI‐II) [16], leech eglin‐C (EGL) [17], *Streptomyces* subtilisin inhibitor (SSI) [18], and marinostatin (MST) [11]). As shown in Table 3, hydrophobic amino acids, such as Leu (ASIP, OMTKY3, and EGL), Met (CI‐2, SSI, and MST), and Phe (TI‐II domain 2) are mainly found at the P1 position since subtilisin BPN′ hydrolyzes the peptide bond at the C‐terminal side of hydrophobic amino acid residues. A basic residue (Arg in TI‐II domain 1) was also adopted at the P1 position of the inhibitors. The S1 binding pocket of subtilisin BPN′ is large enough to accommodate the P1 residue [19]. The Thr residue at the P2 position is highly conserved except for SSI (Pro), possibly because of the formation of a hydrogen bond network with a hydrophilic residue at the P1′ site to enhance the rigidity around the reactive site of inhibitors. However, ASIP has a Cys residue required for the disulfide bond at the P2 site (Cys5). Similar to that in other inhibitors, the Ser residue at the P3 site of ASIP is a small amino acid, which might prevent steric hindrance with subtilisin BPN′ at this position. By contrast, the Phe residue at the P4 site of ASIP is considered to play an important role in the interaction with subtilisin BPN′ because of the presence of the S4 pocket of subtilisin, which is capable of accepting a hydrophobic amino acid, such as Phe (ASIP and MST), Ala (OMTKY3 and TI‐II), and Met (SSI), predominantly through interaction with Tyr104 of subtilisin BPN′. This activity is a notable feature of the substrate‐binding pocket of subtilisin BPN′. In fact, substituting Phe3 at the P4 site of ASIP resulted in a decrease in the inhibitory activity of ASIP. Regarding the P1′ site, substituting Ser7 with Ala resulted in an increase in inhibitory activity and binding affinity for ASIP, whereas substituting Ser7 with Asp diminished the inhibitory activity. By contrast, other inhibitors possess a hydrophilic amino acid at this site excluding SSI (Val), which might form a hydrogen bond network with the relatively conserved Thr residue at the P2 site. These differences between ASIP and other inhibitors could be related to the mechanisms that cause conformational rigidity of the main chain at the reactive site to prevent hydrolysis by a protease. For this purpose, various types of interactions, including disulfide bonds, are observed in proteinaceous inhibitors. ASIP has a disulfide bond connecting Cys5‐Cys10, although only four amino acid residues occur between the two Cys residues, which might lead to a more rigid conformation at the reactive site of ASIP compared to that with other inhibitors. In addition, the clogged gutter mechanism [20] can also explain the strong inhibition by ASIP. ASIP, identified by phage display was found to be one of the good substrates for subtilisin BPN′, and the intramolecular covalent bridge of ASIP connecting P2–P4′ residues tethers the two regions across the peptide bond cleavage site. Thus, even if the peptide bond is cleaved by the protease, the resultant peptides tethered by the disulfide bond would remain in the active site cleft of subtilisin. Among the other sites of the C‐terminal side of the reactive site, substituting Thr12 at the P6′ site of ASIP with a basic amino acid increased the inhibitory activity and binding affinity, but the effects of Ala substitution at the other sites were limited. This finding indicates the possibility of forming electrostatic interactions between a basic amino acid at P6′ of ASIP and an acidic amino acid in subtilisin BPN′, although such a residue has not been identified. In future, structural analyses of mutated ASIP with S7A and T12K substitutions in complex with subtilisin BPN′ will clarify this point and the nucleophilic attack angle of the catalytic serine residue on the P1 reactive site for the arrest of the protease reaction. It will also highlight the importance of interactions at the other sites discussed previously herein. In this study, by combining phage display selection and subsequent mutational analyses, including Ala scanning, we created a peptide inhibitor with 12 residues that bind and inhibit subtilisin BPN′ with a *K*_i_ value of 0.3 nm. Its length might be among the shortest observed for peptides composed of common amino acids that show strong inhibitory activity with *K*_i_ less than 1 nm. Such a short length is associated with the formation of the disulfide bond and might be due to the presence of a specific P4–S4 interaction in addition to the P1–S1 interaction in well‐known proteinaceous inhibitors. As described, further structural analyses of the complex will clarify these points, and such analyses will also provide valuable insights for the further development of a functional peptide inhibitor.

**Table 3:** Proteinaceous subtilisin inhibitors. Amino acid residue lengths, sequences from the P4 to P4′ positions, and *K*_i_ values against subtilisin BPN′ or Carlsberg of the inhibitors are listed. The most conserved residues at each position are shown in bold font. Residue numbers at the P1 position are indicated by superscript numbers. CI‐2, hiproly barley chymotrypsin inhibitor 2; EGL, leech eglin‐C; MST, marinostatin; OMTKY3, turkey ovomucoid third domain; S7A + T12K, double mutant of ASIP (S7A and T12K); SSI, *Streptomyces* subtilisin inhibitor; TI‐II (D1: domain 1; D2: domain 2), wound‐induced tomato protease inhibitor 2.

	Length	P4	P3	P2	P1	P1′	P2′	P3′	P4′	*K*_i_ (nM)	Ref.
ASIP	12	Phe	Ser	Cys	**Leu** ^6^	Ser	Glu	Gly	Cys	13.0	This study
S7A + T12K	12	Phe	Ser	Cys	**Leu** ^6^	Ala	Glu	Gly	Cys	0.30	This study
OMTKY3	56	**Ala**	**Cys**	**Thr**	**Leu** ^18^	**Glu**	**Tyr**	**Arg**	**Pro**	0.023	[21]
CI‐2	64	Ile	Val	**Thr**	**Met** ^59^	**Glu**	**Tyr**	**Arg**	Ile	0.003	[15]
TI‐II (D1)	123	**Ala**	**Cys**	**Thr**	Arg^5^	**Glu**	Cys	Gly	Asn	9^a^	[22]
TI‐II (D2)		**Ala**	**Cys**	**Thr**	Phe^62^	Asn	Cys	Asp	**Pro**		
EGL	70	Pro	Val	**Thr**	**Leu** ^45^	Asp	Leu	**Arg**	Tyr	0.15^a^	[23]
SSI	113	Met	**Cys**	Pro	**Met** ^73^	Val	**Tyr**	Asp	**Pro**	0.005	[24]
MST	12	Phe	Ala	**Thr**	**Met** ^4^	Arg	**Tyr**	Pro	Ser	1.5	[11]

^a^ Inhibitory activity against subtilisin Carlsberg.

## Conflict of interest

The authors declare no conflict of interest

## Author contributions

SK conceived and supervised the study; SK designed the experiments; MS, AW, and YM performed the experiments; KI, MS, AW, YM, AN, and SK analyzed the data; KI, AN, and SK wrote the manuscript; KI, AN, and SK made the manuscript revision.

## Data Availability

The data that support the findings of this study are available from the corresponding author [akira.nakamura@gakushuin.ac.jp] upon reasonable request.
